# Microbiome of *Trichodesmium* Colonies from the North Pacific Subtropical Gyre

**DOI:** 10.3389/fmicb.2017.01122

**Published:** 2017-07-06

**Authors:** Mary R. Gradoville, Byron C. Crump, Ricardo M. Letelier, Matthew J. Church, Angelicque E. White

**Affiliations:** ^1^College of Earth, Ocean and Atmospheric Sciences, Oregon State University Corvallis, OR, United States; ^2^Flathead Lake Biological Station, University of Montana MT, United States

**Keywords:** *Trichodesmium*, marine microbiome, *nifH* diversity, heterotrophic marine diazotrophs, metagenomics, 16S rRNA, nitrogen fixation

## Abstract

Filamentous diazotrophic Cyanobacteria of the genus *Trichodesmium*, often found in colonial form, provide an important source of new nitrogen to tropical and subtropical marine ecosystems. Colonies are composed of several clades of *Trichodesmium* in association with a diverse community of bacterial and eukaryotic epibionts. We used high-throughput 16S rRNA and *nifH* gene sequencing, carbon (C) and dinitrogen (N_2_) fixation assays, and metagenomics to describe the diversity and functional potential of the microbiome associated with *Trichodesmium* colonies collected from the North Pacific Subtropical Gyre (NPSG). The 16S rRNA and *nifH* gene sequences from hand-picked colonies were predominantly (>99%) from *Trichodesmium* Clade I (i.e., *T. thiebautii*), which is phylogenetically and ecologically distinct from the Clade III IMS101 isolate used in most laboratory studies. The bacterial epibiont communities were dominated by Bacteroidetes, Alphaproteobacteria, and Gammaproteobacteria, including several taxa with a known preference for surface attachment, and were relatively depleted in the unicellular Cyanobacteria and small photoheterotrophic bacteria that dominate NPSG surface waters. Sequencing the *nifH* gene (encoding a subcomponent of the nitrogenase enzyme) identified non-*Trichodesmium* diazotrophs that clustered predominantly among the Cluster III *nifH* sequence-types that includes putative anaerobic diazotrophs. *Trichodesmium* colonies may represent an important habitat for these Cluster III diazotrophs, which were relatively rare in the surrounding seawater. Sequence analyses of *nifH* gene transcripts revealed several cyanobacterial groups, including heterocystous *Richelia*, associated with the colonies. Both the 16S rRNA and *nifH* datasets indicated strong differences between *Trichodesmium* epibionts and picoplankton in the surrounding seawater, and also between the epibionts inhabiting *Trichodesmium* puff and tuft colony morphologies. Metagenomic and 16S rRNA gene sequence analyses suggested that lineages typically associated with a copiotrophic lifestyle comprised a large fraction of colony-associated epibionts, in contrast to the streamlined genomes typical of bacterioplankton in these oligotrophic waters. Additionally, epibiont metagenomes were enriched in specific genes involved in phosphate and iron acquisition and denitrification pathways relative to surface seawater metagenomes. We propose that the unique microbial consortium inhabiting colonies has a significant impact on the biogeochemical functioning of *Trichodesmium* colonies in pelagic environments.

## Introduction

The filamentous, dinitrogen (N_2_)-fixing (diazotrophic) cyanobacterium *Trichodesmium* provides a major source of bioavailable nitrogen (N) to the oligotrophic subtropical and tropical oceans (Karl et al., 2002; Capone et al., 2005). *Trichodesmium* abundances and N_2_ fixation rates have been integral components of global N_2_ fixation estimates and models (e.g., Coles et al., 2004; Mahaffey et al., 2005); thus, an accurate understanding of the physiology and ecology of this genus is crucial. Most *Trichodesmium* laboratory studies have used a single isolate, *T. erythraeum* strain IMS101, grown in culture with minimal heterotrophic bacteria. In contrast, natural *Trichodesmium* populations are composed of species from four known phylogenetically distinct clades (Hynes et al., 2012), which can vary in physiological traits, such as carbon (C) affinity and phosphonate biosynthesis (Dyhrman et al., 2009; Hutchins et al., 2013). Furthermore, in nature they are commonly found associated with attached microorganisms (Borstad and Borstad, 1977). The diversity of this complex community likely affects the overall functioning of colonies (Gradoville et al., 2014), yet few studies have examined the ecology of *Trichodesmium* species and associated epibionts (although see Hmelo et al., 2012; Rouco et al., 2016).

*Trichodesmium* cells exist as free filaments or aggregate colonies (Letelier and Karl, 1996) with varying morphologies, namely spherical “puffs” and fusiform “tufts.” These colonies have been reported to maintain an active and diverse assemblage of attached organisms, including bacteria, eukaryotic phytoplankton, protozoa, fungi, and copepods (Borstad and Borstad, 1977; Sheridan et al., 2002). *Trichodesmium* colonies constitute a favorable environment for associated epibionts by providing buoyancy (Walsby, 1992), elevated concentrations of dissolved organic N (Capone et al., 1994), and a substrate for attachment (O'Neil, 1998). Recent studies using 16S rRNA gene sequencing have shown that *Trichodesmium*-associated bacterial epibionts include surface-associated taxa (Hmelo et al., 2012) and that selective processes appear to drive epibiont community structure (Rouco et al., 2016).

Less is known about how associated microorganisms affect the functioning of the *Trichodesmium* holobiont. *Trichodesmium* colonies appear to be hotspots for microbial activity: hydrolytic enzyme activities are elevated within colonies (Stihl et al., 2001; Sheridan et al., 2002) and a metatranscriptome from *Trichodesmium* bloom material recovered more transcripts from associated organisms than from *Trichodesmium* cells (Hewson et al., 2009). Microbial processes carried out by associated microorganisms have the potential to influence rates of N_2_ or C fixation. For instance, quorum sensing by associated bacteria can increase alkaline phosphatase activity within colonies (Van Mooy et al., 2011), which could stimulate *Trichodesmium* dissolved organic phosphorus utilization, thereby increasing N_2_ fixation rates when phosphate is limiting. Likewise, specific epibiont bacteria may secrete siderophores, chelating iron which could subsequently become bioavailable to *Trichodesmium* after photodegradation (Roe et al., 2012). Associated microorganisms may also directly contribute to the fixation of C and/or N_2_. Phototrophs including filamentous Cyanobacteria (Siddiqui et al., 1992) and diatoms (Borstad and Borstad, 1977) have historically been observed within *Trichodesmium* colonies. More recently, heterocystous cyanobacterial diazotrophs have been observed within *Trichodesmium* colonies (Momper et al., 2015) and *nifH* genes (encoding a subcomponent of the nitrogenase enzyme) phylogenetically clustering among facultative anaerobes and aerobic heterotrophic bacteria have been retrieved from *Trichodesmium* colonies (Gradoville et al., 2014). The degree to which these associated diazotrophs contribute to bulk colony N_2_ fixation rates is unknown.

Here, we examine the microbiome associated with *Trichodesmium* colonies collected from the North Pacific Subtropical Gyre (NPSG). We used a combined approach of high-throughput 16S rRNA and *nifH* gene sequencing, metagenomics, and ^13^C and ^15^N_2_ fixation assays to survey the diversity of the *Trichodesmium* holobiont, test for the presence and activity of non-*Trichodesmium* colony-associated diazotrophs, and explore the functional potential of the colonies. We compare the colony-associated microbiome to the microbial community structure and metagenomic composition of surrounding seawater, revealing diverse and unique microbial structure and functional potential associated with *Trichodesmium* colonies.

## Methods

### Sample collection

**Figure 1:**
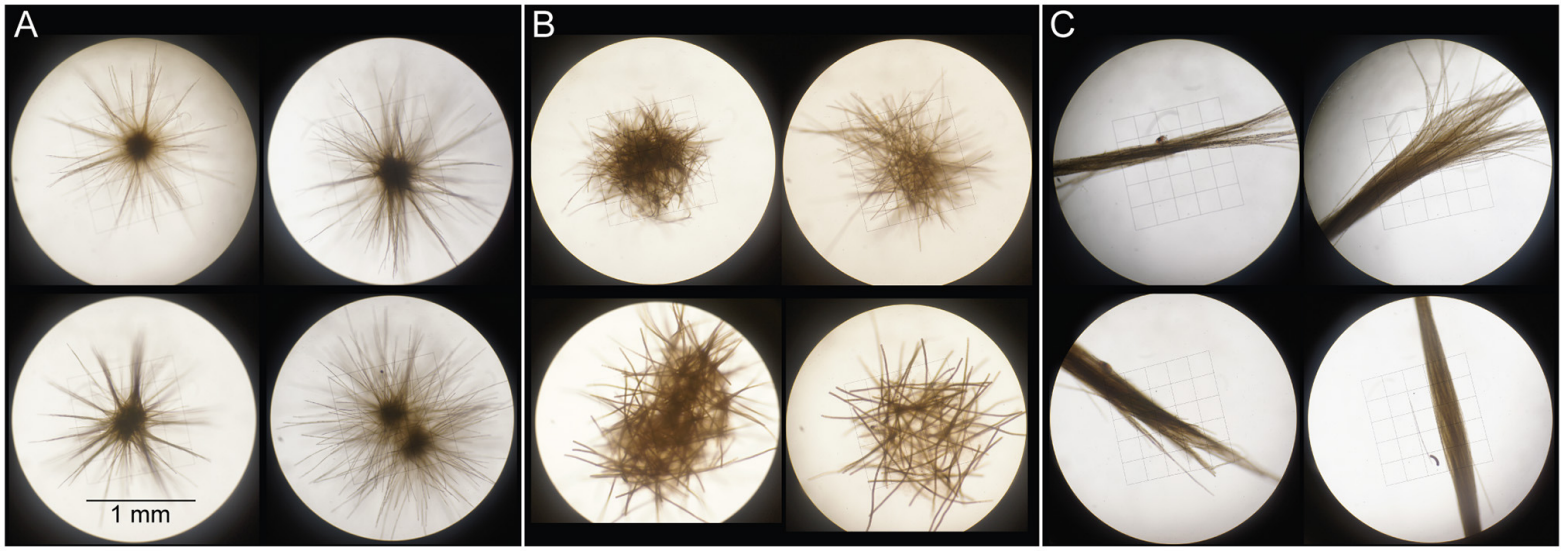
Examples of *Trichodesmium* colonies sorted into the morphological classes “radial puffs” **(A)**, “non-radial puffs” **(B)**, and “tufts” **(C)** on 23 Mar 2014. On all other collection days, “puffs” designate mixtures of morphotypes **(A,B)**, “tufts” designate morphotype **(C)**, and “mixed” designates mixtures of all morphotypes.

Samples were collected in March 2014 aboard the R/V *Kilo Moana* at Stn. ALOHA (A Long-term Oligotrophic Habitat Assessment; 22.45°N, 158°W), an open-ocean field site ~100 km north of Oahu (Table 1). *Trichodesmium* colonies were collected using a 202 μm plankton net which was hand-towed at <2 km h^−1^ through near-surface waters (<10 m depth) for 10–15 min. Once recovered, colonies were isolated using an inoculating loop and rinsed twice with 0.2 μm-filtered surface seawater prior to all analyses. Colonies were sorted into morphological classes of spherical “puffs” (further divided into “radial puffs” and “non-radial puffs” on 23 Mar), fusiform “tufts,” and “mixed” morphologies (Figure 1), and either filtered for subsequent extraction of DNA and RNA or used for C and N_2_ fixation measurements. Additionally, bulk seawater from 25 m depth was collected for comparison with *Trichodesmium* colony DNA. Seawater samples were collected using sampling bottles attached to a CTD (conductivity, temperature, depth) rosette, and subsampled into 4 L acid-washed, MilliQ-rinsed polycarbonate bottles prior to filtration.

**Table 1:** Summary and environmental conditions for sampling dates during a March 2014 cruise at Stn. ALOHA.

**Date**	**SST (°C)**	**Chl (μg L^−1^)**	**Morphologies used**	**Measurements**	**C fixation rate (nmol C μmol C^−1^ h^−1^)**	**N_2_ fixation rate (nmol N μmol C^−1^ h^−1^)**
12 Mar	24.2	0.16	25 m seawater only	DNA	ND	ND
13 Mar	24.2	0.15	Puff, tuft	DNA	ND	ND
14 Mar	24.1	0.16	Puff, tuft	DNA, RNA, rates	Puff: 7.9 (1.2) Tuft: 7.2 (0.6)	Puff: 0.02 (0.003) Tuft: 0.01 (0.004)
18 Mar	23.8	0.25	25 m seawater only	DNA	ND	ND
20 Mar	23.8	0.22	Mixed	DNA, rates	9.1 (1.8)	0.09 (0.05)
21 Mar	23.8	0.21	Puff, tuft	DNA, RNA	ND	ND
22 Mar	23.8	0.20	Mixed	DNA, RNA, rates	10.1 (1.9)	0.14 (0.08)
23 Mar	23.8	0.18	Mixed	Rates	9.7 (1.5)	0.17 (0.05)
23 Mar	23.9	0.11	R puff, NR puff, tuft	DNA, microscopy	ND	ND

*All samples were collected pre-dawn, with the exception of 23 Mar 2014, when samples were collected mid-afternoon. Sea surface temperature (SST) and surface chlorophyll fluorescence (Chl) were measured at 25 m depth using conductivity-temperature-depth sensors. Rates represent averages of duplicate incubation bottles, with standard deviations in parentheses. R denotes radial; NR denotes non-radial (see Figure 1); ND indicates not determined*.

### Carbon and nitrogen fixation rates

Carbon (C) and dinitrogen (N_2_) fixation rates were measured using the ^13^C method of Legendre and Gosselin (1997) and a modification of the ^15^N_2_ uptake method of Montoya et al. (1996) to avoid delayed bubble dissolution (Mohr et al., 2010; Wilson et al., 2012). ^15^N_2_ was added to incubations via ^15^N_2_-enriched seawater, which was prepared onshore ~1 week prior to departure using Stn. ALOHA surface seawater according to the methods of Wilson et al. (2012). Briefly, seawater was 0.2 μm-filtered and degassed, then dispensed into gas-tight 3 L PTFE bags (Welch Fluorocarbon); 12.9 mL^−L^ ^15^N_2_ gas (Cambridge Isotopes, 99%) was injected into the bag, which was manually agitated to facilitate dissolution. This ^15^N_2_-enriched seawater was dispensed into glass serum bottles, which were capped, crimped, and stored at 4°C until use. The ^15^N_2_ content of enriched seawater was validated via Membrane Inlet Mass Spectrometry according to the methods of Böttjer et al. (2017).

For the incubations, 20–30 colonies were transferred into 37 mL glass serum bottles filled with 0.2 μm-filtered surface seawater. Samples were spiked with 4 mL ^15^N_2_-enriched seawater and 0.5 mL of 48 mmol L^−1^ ^13^C bicarbonate stock, and bottles were topped off with filtered seawater, capped with Viton septa and aluminum caps, and crimp-sealed. Samples were incubated from dawn to dusk (~12 h) in flow-through deckboard incubators with blue acrylic shading used to simulate ~60% of the sea-surface irradiance. Incubations were terminated by gentle filtration onto 25 mm diameter pre-combusted glass fiber filters (Whatman GF/F). Additionally, 20–30 colonies were preserved for duplicate δ^15^N natural abundance (time-zero samples) after each net tow. Filters were flash-frozen and shipped to Oregon State University, where they were dried at 60°C overnight and packed into tin and silver capsules. Isotopic composition and masses of particulate N and C were measured with an isotope ratio mass spectrometer at Oregon State University. Fixation rates were calculated according to Montoya et al. (1996) and normalized to particulate C concentrations; thus, N_2_ fixation rates are expressed as nmol N μmol C^−1^ d^−1^ rather than nmol N_2_ L^−1^ d^−1^.

### Nucleic acid extraction, amplification, and sequencing

For samples used for subsequent extraction of DNA and RNA, 20–30 *Trichodesmium* colonies were transferred into filtered seawater and gently filtered onto 25 mm diameter, 0.2 μm polyethersulfone Supor filters (Pall Corporation). Samples for subsequent extraction of planktonic DNA from 25 m seawater were filtered onto 0.2 μm Supor filters using a peristaltic pump. Filters were placed into empty microcentrifuge tubes (DNA) or microcentrifuge tubes containing 0.5 mL RNAlater (RNA), flash-frozen, transported in liquid N_2_ to Oregon State University, and stored at −80°C until analysis. DNA was extracted using the DNeasy Plant MiniKit (Qiagen), with a modified protocol to include a freeze-fracture step and Proteinase K treatment. RNA was extracted using the RNeasy MiniKit (Qiagen) according to manufacturer instructions, with additional steps for cell disruption through flash-freezing and bead-beating filters in mixtures of 500 μL RLT buffer, 5 μL β-mercaptoethanol, and 200 μL of mixed 0.1 mm and 0.5 mm glass beads (Biospec products). Possible carry-forward DNA contamination was minimized from RNA extracts by using the Turbo DNA-free kit (Ambion), and extracts were quantified using a Qubit RNA HS Assay kit (Invitrogen). Complimentary DNA (cDNA) was synthesized using the SuperScript III First-Strand kit (Invitrogen) according to the manufacturer's instructions, using the nifH3 gene-specific primer (Zani et al., 2000). DNA and cDNA were quantified with the Quant-iT PicoGreen dsDNA Assay Kit (Invitrogen) using a MicroMax 384 plate reading fluorometer, and extracts were stored at −20 or −80°C.

The polymerase chain reaction (PCR) was used to amplify a portion of the 16S rRNA gene, targeting the entire prokaryotic community (DNA samples only), and the *nifH* gene, targeting diazotrophs (for both DNA and cDNA). All PCR reactions were performed using a Veriti (Applied Biosystems) or DNAEngine (BioRad) thermocycler and 10 or 20 μL reaction volumes. 16S rRNA PCR consisted of 1X HotMasterMix (5 PRIME), 1 μL DNA extract, and 5 pmol 515f (GTGCCAGCMGCCGCGGTAA) and 806r (GGACTACHVGGGTWTCTAAT) primers (Caporaso et al., 2010) which were modified to include Illumina adapters and dual-index barcodes as described by Kozich et al. (2013). Thermal cycling conditions for 16S rRNA gene amplifications were: 94°C for 3 min, followed by 30 cycles of 94°C for 45 s, 50°C for 60 s, and 72°C for 90 s, with a final 72°C extension for 10 min.

The *nifH* gene was amplified using nested degenerate *nifH* primers (Zehr and McReynolds, 1989; Zani et al., 2000). The first round contained 1X PCR buffer, 0.1U Platinum High Fidelity *Taq* polymerase (Invitrogen), 200 μmol L^−1^ dNTPs, 3% BSA, 4 mmol L^−1^ Mg^2+^, 1 μL DNA or cDNA, and 1 μmol L^−1^ nifH1 and nifH2 primers (Simon et al., 2014). Reaction conditions were: 94°C for 7 min, followed by 30 cycles of 94°C for 1 min, 57°C for 1 min, and 72°C for 1 min, and a final 72°C extension for 7 min. The second round of *nifH* PCR used the same components and thermocycling conditions as the first round, except the DNA extract was replaced with 1 μL of the amplified product generated during the first round PCR reaction, and custom primers were used, consisting of gene-specific sites (nifH3 and nifH4), dual-indexed barcodes, Illumina linkers, and a sequencing primer binding region, similar to those described by Kozich et al. (2013; Table S1). PCR negative controls and filter blank samples were included in PCR reactions.

Triplicate PCR reactions were visualized by gel electrophoresis, then pooled and quantified as above. Samples were only sequenced if they had three successful PCR reactions, except for PCR negative controls and filter blanks, which were sequenced despite the absence of visual gel bands after amplification. 16S rRNA and *nifH* gene amplicons were pooled to equimolar concentrations, cleaned using both the UltraClean PCR (MoBio) and AMPure XP Bead cleanup kits, and sequenced at Oregon State University using MiSeq Standard v.3, 2 × 300 bp paired-end sequencing.

Metagenomes were constructed from two *Trichodesmium* puff DNA samples (Figure 1). Libraries were constructed using an Illumina Nextera XT library prep kit, and cleaned using the AMPure XP Bead cleanup kits. Samples were sequenced on an Illumina MiSeq using a v.3 MiSeq Reagent Kit and a 2 × 300 bp paired-end protocol. Metagenome library preparation, cleaning, and sequencing were carried out by the Oregon State University Center for Genome Research and Biocomputing Center.

### Bioinformatic analyses

Sequence reads from 16S rRNA gene amplicons, *nifH* gene amplicons, and metagenomes were demultiplexed using the Illumina MiSeq Reporter (MSR) version 2.5.1. For 16S rRNA gene sequences, primers were also removed using MSR. The majority of 16S rRNA gene paired-end reads were merged and screened for quality, retaining sequences between 245 and 254 bp with no ambiguities using mothur (Schloss et al., 2009). For a subset of 16S rRNA gene samples, only forward reads were used for phylogenetic analyses due to the poor quality of reverse reads. The reverse primer was trimmed from forward reads, and reads with ambiguities, homopolymers (>8 bp) or poor quality (average score <25 or any score <20) were removed using mothur. Finally, forward reads with lengths between 245 and 254 bp were retained and combined with the paired-end sequences for subsequent analyses. Singletons were removed, operational taxonomic units (OTUs) were clustered at 97% nucleotide sequence similarity, and a chimera check was performed with the Gold ChimeraSlayer reference database using USearch (Edgar, 2010). After quality control procedures, two of four PCR negative controls, and one of two triplicate-pooled filter blank samples retained a small number of sequences (8, 1496, and 21 sequences, respectively). Taxonomy was assigned in QIIME using the Silva v123 reference database, and sequences classified as chloroplasts, mitochondria, Archaea, Eukaryota, or an unknown domain were removed. Sequences were subsampled to 7,011 sequences per sample, resulting in near-saturation for most rarefaction curves (Figure S1). Negative control and filter blank samples contained less sequences than this cutoff, and were thus excluded from further analyses. Nonmetric multidimensional scaling analyses (NMDS, via Bray-Curtis similarity) and alpha diversity metric calculations were performed using QIIME (Caporaso et al., 2010). This same procedure was performed on reduced datasets containing *Trichodesmium* OTUs only (excluding 25 m seawater samples; rarefied to 2,248 sequences per sample) and containing all non-*Trichodesmium* OTUs (rarefied to 3,128 sequences per sample).

For *nifH* amplicons, though both forward and reverse barcodes were used for demultiplexing, only forward reads were used for phylogenetic analyses due to the poor quality of reverse reads. Reads with ambiguities, poor quality, or homopolymers were discarded. Forward primers were removed, sequences were trimmed to 244 bp, and OTUs were clustered at 97% nucleotide sequence similarity using USearch with a *de novo* chimera checker (Edgar, 2010). OTUs containing chimeras, frameshifts, and non-*nifH* sequences were removed. The three PCR negative controls contained no *nifH* sequences after these quality control procedures, while two of five filter blank samples contained a small number of sequences (1 and 330 sequences). Sequences were subsampled to 9,651 sequences per sample, saturating most rarefaction curves (Figure S1). Both filter blank samples contained less sequences than this cutoff, and were excluded from further analyses. The *nifH* OTUs were translated and phylogenetically classified into *nifH* gene clusters (Zehr et al., 2003) via BLAST-p similarity to a reference database of *nifH* gene sequences (http://www.jzehrlab.com/#!nifh-database/c1coj). Sequences were termed “undefined” if they had equal amino acid similarity to sequences from multiple *nifH* gene sequence-types. BLASTn searches of the National Center for Biotechnology Information (https://blast.ncbi.nlm.nih.gov) were also performed for select *nifH* and 16S rRNA gene OTUs.

Metagenome sequences were demultiplexed using the Illumina MSR version 2.5.1. All further processing steps were performed for the two *Trichodesmium* colony metagenomes and a metagenome previously constructed from Stn. ALOHA surface seawater DNA (15 m depth, 0.2 μm pore-size filter) on 30 July 2015 (Wilson et al., under review; NCBI BioProject accession PRJNA358725, BioSample S37C001). Raw reads were assembled separately for each sample using MEGAHIT (Li et al., 2015). Assemblies were uploaded to the Joint Genome Institute Genomes Online Database (https://gold.jgi.doe.gov/), where coding sequences (CDS) were predicted and annotated to the Kyoto Encyclopedia of Genes and Genomes (KEGG, Kanehisa and Goto, 2000; Huntemann et al., 2015). Metagenome sequences were processed according to the methods of Nalven (2016). Sequence reads were then trimmed for quality using seqtk (https://github.com/lh3/seqtk) and mapped back to CDS using Bowtie 2 (Langmead and Salzberg, 2012). Counts (one for single reads and two for paired reads mapped), CDS lengths, and alignment lengths were extracted using SAMtools (Li et al., 2009), and counts were normalized to account for length of reads and length of CDS (Wagner et al., 2012). Counts within KEGG ortholog groups (KO) were summed and normalized as counts per million mapped to KO-annotated contigs [Genes Per Million (GPM), Wagner et al., 2012] and as counts per million mapped to KO-annotated contigs of known function (designated GPMK). GPM counts were used to analyze overall taxonomy, while GPMK were used for functional analyses. Counts from each KO were also divided into categories assigned to Cyanobacteria (assumed to be predominantly *Trichodesmium*) and non-Cyanobacteria. Details on the assembly and annotation of each sample are provided in Table 2.

**Table 2:** Summary of metagenome assembly, annotation, and mapping.

	***Trichodesmium* non-radial puff colonies**	***Trichodesmium* radial puff colonies**	**Stn. ALOHA surface seawater**
Illumina paired-end reads	13,294,194	8,629,462	14,035,332
Contigs assembled	1,771,587	1,341,086	444,296
Weighted-average contig length (N50^a^)	315 bp	301 bp	539 bp
Contigs annotated to KO	454,684	290,117	330,104
Contigs annotated (%)	25.7	21.6	74.3
Counts mapped to KO	6,446,495	3,743,920	3,669,469
Counts mapped to KO of known function^b^	3,664,674	2,116,212	2,550,585
Genomes per million genes^b^^,^^c^	417 (478)	423 (476)	823
KO of known function (%)	56.8 (57.9)	56.5 (59.6)	69.5

*Trichodesmium samples were collected 23 Mar 2014; seawater sample was collected 30 July 2015. Parenthetical values represent only those KO assigned to non-Cyanobacteria*.

a *N50 values were generated by MEGAHIT*.

b *Length-corrected counts*.

c *Average GPM from 29 KOs previously identified as single-copy genes (Nayfach and Pollard, 2015, Table S4)*.

All raw sequences are available from NCBI (accession SRP078449). Assemblies and annotation data are available from IMG/M ER (http://img.jgi.doe.gov/mer; Taxon OIDs 3300009572, 3300009536, and 3300010936).

### Statistical analyses

Two-way ANOVA with subsequent Tukey Honest Significant Difference (HSD) *post-hoc* tests were used to test the effect of day and sample type on N_2_ fixation rates and alpha diversity metrics. The Welch Two Sample *t*-test was used to test for differences in the relative proportion of puff and tuft sequences in dominant OTUs, using the Bonferroni correction for multiple comparisons. Both ANOVA and *t*-tests were performed using the program R (http://www.r-project.org/). One-way ANOSIM tests were used to test for significant differences in community structure among sample types, using the program PRIMER. Detection limits for N_2_ fixation rate measurements were calculated using standard propagation of errors via the observed variability between replicate samples as described by Gradoville et al. (2017) (Table S2).

## Results

### Carbon and nitrogen fixation rates

Shipboard incubation experiments showed that *Trichodesmium* colonies were actively fixing N_2_ and C. Biomass-normalized ^15^N_2_ fixation rates ranged from 0.24 to 4.16 nmol N μmol C^−1^ d^−1^ (Table 1); all rates were above detection limits (Table S2). Biomass-normalized ^13^C fixation rates ranged from 173 to 243 nmol C μmol C^−1^ d^−1^ (Table 1). Both ^15^N_2_ and ^13^C rates were normalized to C content rather than colony number due to the known variability in the size of *Trichodesmium* colonies (Letelier and Karl, 1996). These ranges are similar to previously reported *Trichodesmium* colony-specific (Lomas et al., 2012) and C-specific (Gradoville et al., 2014) N_2_ and C fixation rates. N_2_ fixation rates varied by day of sampling (two-way ANOVA, *p* < 0.01) but not by morphology (*p* > 0.05). C fixation rates did not vary by either day or morphology (two-way ANOVA, *p* > 0.05).

### *Trichodesmium* species diversity

**Figure 2:**
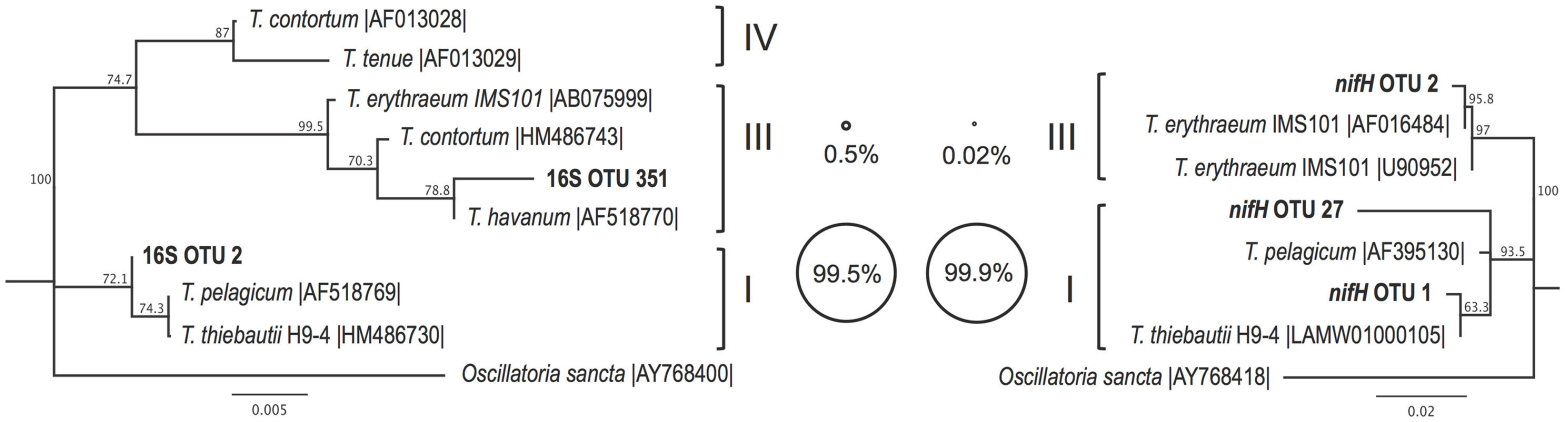
Neighbor joining phylogenetic trees depicting the relationships between *Trichodesmium* OTUs (97% nucleotide similarity) from partial 16S rRNA **(left)** and partial *nifH* **(right)** gene sequences, together with reference sequences from cultivated representatives (accession numbers given). Major *Trichodesmium* clades (Lundgren et al., 2005) are shown in Roman numerals. Bubble plots depict the percentage of *Trichodesmium* DNA sequences from this study which group with each clade, according to partial 16S rRNA **(left)** and partial *nifH* **(right)** amplicon datasets. Bootstrap values (1,000 replicates) of >50% are provided. Scale bars represent nucleotide substitutions per site.

**Figure 3:**
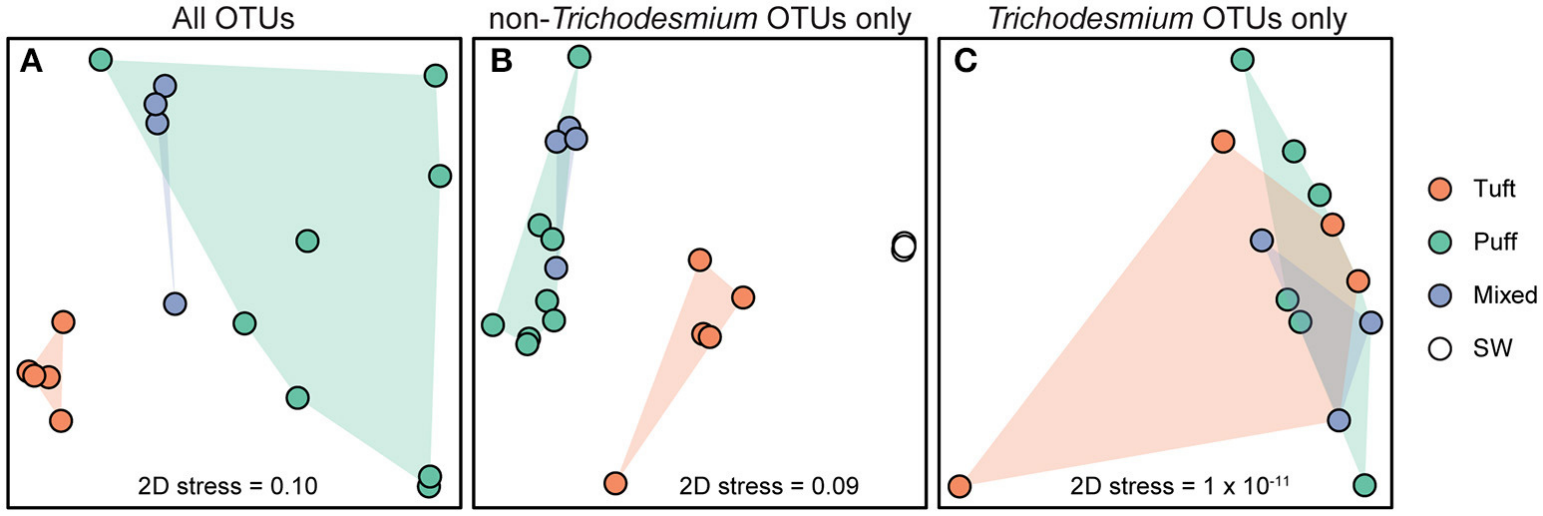
Non-metric multi-dimensional scaling (NMDS) plots derived from the Bray–Curtis dissimilarity matrix of 16S rRNA OTUs from **(A)** all *Trichodesmium* colony sample OTUs (7011 sequences per sample), **(B)** *Trichodesmium* colony and surface seawater samples excluding *Trichodesmium* OTUs (3128 sequences per sample), and **(C)** *Trichodesmium* colony samples excluding non-*Trichodesmium* OTUs (2248 sequences per sample). Each point represents an individual sample. Colors represent sample type [tuft colonies, puff colonies, mixed colonies, and bulk seawater (SW) from 25 m].

The *Trichodesmium* species diversity within our samples was assessed via PCR amplification and sequencing of 16S rRNA and *nifH* genes. Sequences from both genes indicate that *Trichodesmium* Clade I (e.g., *T. thiebautii*) dominated our samples, with Clade III (e.g., *T. erythraeum*) representing <1% of *Trichodesmium* sequences (Figure 2). The 16S rRNA gene dataset contained 2 OTUs classified as *Trichodesmium*, with the most abundant OTU classified as Clade I (16S OTU 2, 99.5% of *Trichodesmium* 16S rRNA sequences). Likewise, 2 of the 3 *Trichodesmium nifH* gene OTUs (*nifH* OTU 1 and *nifH* OTU 27) were classified as Clade I and together comprised 99.9% of the *Trichodesmium nifH* gene sequences (Figure 2). Puff, tuft, and mixed morphology samples from 16S rRNA and *nifH* genes all contained >99% Clade I *Trichodesmium* sequences (Table S3), and the *Trichodesmium* community structure did not vary by morphology (ANOSIM R = −0.042, *p* = 0.6; Figure 3C). No sequences from the 16S rRNA or *nifH* gene datasets were classified as *Trichodesmium* Clade II or Clade IV.

### Microbial diversity via 16S rRNA gene amplicons

**Figure 4:**
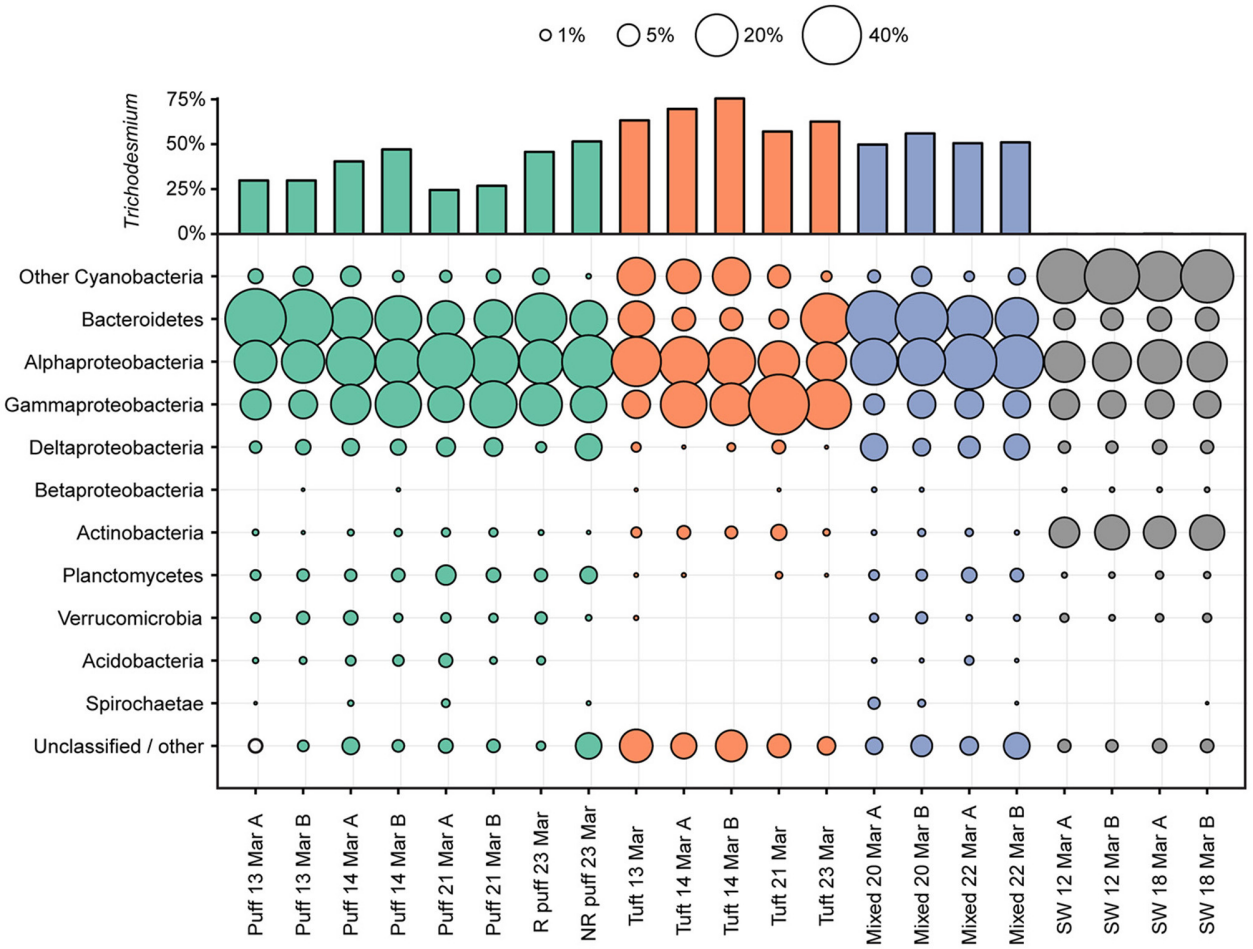
Percentages of partial 16S rRNA gene sequences assigned to bacterial taxa. Percentages of sequences assigned to *Trichodesmium* are displayed in the upper bar plot. The area plot displays the percentages of non-*Trichodesmium* sequences assigned to other bacterial taxa. Color indicates the sample type/morphology [green, red, blue, and gray for puff colonies, tuft colonies, mixed colonies, and bulk 25 m seawater (SW), respectively]. R denotes radial and NR denotes non-radial puff morphologies (see Figure 1).

The microbial diversity of the *Trichodesmium* microbiome was assessed using high-throughput sequencing of partial 16S rRNA genes from 17 *Trichodesmium* colony samples and 4 surface seawater samples for comparison. *Trichodesmium* sequences represented 24–75% of 16S rRNA amplicons from colony samples; the remaining 25–76% of sequences corresponded to associated bacteria, termed epibionts (though it is possible that a subset of these organisms were endobionts). The most abundant epibiotic taxa belonged to Bacteroidetes (Cytophagia, Sphingobacteriales, and Flavobacteriales), Alphaproteobacteria (predominantly Rhodobacteriales, Rhodospirillales, and Rhizobiales), and Gammaproteobacteria (e.g., *Marinicella* sp., *Alteromonas* sp., Oceanospirillales) (Figure 4, Figure S2, Table S3). Even at broad phylum- and class-level taxonomic groupings, the *Trichodesmium* epibiont community differed from the bacterial community in the surrounding seawater: all colonies were relatively enriched in Bacteroidetes, and puff and mixed colony samples were enriched in Acidobacteria and Deltaproteobacteria, compared to the surrounding seawater (Figure 4). Additionally, some of the most abundant taxa in NPSG near-surface seawater samples, including the Cyanobacteria *Prochlorococcus* sp., and *Synechococcus* sp., the Actinobacteria *Actionomarina* sp., and marine groups AEGEAN-169, SAR11, SAR86, and SAR116, were relatively depleted or absent in *Trichodesmium* colony samples (Figure 4, Figure S2). At the 97% identity level, *Trichodesmium* colonies and seawater samples had few dominant epibiont OTUs (OTUs containing >1% non-*Trichodesmium* sequences in samples of either morphology) in common (Figure S2). NMDS analyses provide further evidence that the community structure of the epibionts was distinct from that of the surrounding seawater, and also illustrate greater dissimilarity among *Trichodesmium* samples than among surface seawater samples (ANOSIM R = 0.852, *p* = 0.001; Figure 3B).

The *Trichodesmium* epibiont community varied with colony morphology. *Trichodesmium* colonies with puff morphology (*n* = 8 samples) contained a smaller fraction of *Trichodesmium* sequences (24–51% *Trichodesmium* 16S rRNA), and thus a larger fraction of epibiont sequences, than tuft morphologies (*n* = 5 samples; 57–75% *Trichodesmium* 16S rRNA) (Figure 4). The epibiont communities of puff colonies contained a larger fraction of Bacteroidetes (including Cytophagia and Saprospiraceae) and Deltaproteobacteria (including Desulfuromonadales) than tuft colonies (Figure 4, Figure S2). Tuft colonies contained a larger fraction of non-*Trichodesmium* Cyanobacteria (predominantly *Limnothrix*) and Gammaproteobacteria (including Alteromonadaceae, Oleiphilaceae, and Piscirickettsiaceae) than puff colonies. There were also differences between puff and tuft colony epibionts at the OTU level: over half of the most abundant *Trichodesmium* OTUs had significantly different relative abundances between the two morphotypes (Figure S2). NMDS analyses demonstrated that the overall epibiont community structure varied by morphology, with puff colonies clustering separately from tuft colonies (Figure 3).

Alpha diversity metrics were calculated from 16S rRNA gene OTUs at 97% identity (Table 3). Both diversity (Shannon) and species richness (Chao1) varied by sample type (i.e., seawater or morphology) and by day of sampling (*p* < 0.05, two-way ANOVA). Species richness did not vary among *Trichodesmium* morphologies (Tukey HSD *p* > 0.05), but all morphotypes had significantly lower (by a factor of ~2) species richness than surface seawater samples (Tukey HSD *p* ≤ 0.001). Diversity was higher in *Trichodesmium* puff samples and mixed morphology samples than in tuft samples (Tukey HSD *p* < 0.001). *Trichodesmium* samples of all morphotypes had lower diversity than seawater samples (Tukey HSD *p* < 0.05); however, when excluding *Trichodesmium* OTUs, diversity in samples of all *Trichodesmium* morphotypes were not significantly different from seawater (Tukey HSD *p* > 0.05). Thus, the *Trichodesmium* epibiont community had lower species richness, but insignificant differences in evenness, compared to seawater.

**Table 3:** Diversity and species richness estimates.

	**Sample type**	**Diversity (Shannon)**	**Species richness (Chao1)**
All OTUs	Puff	4.5 (0.8)	268 (70)
	Tuft	2.5 (0.5)	208 (58)
	Mixed	3.8 (0.2)	263 (15)
	SW	5.1 (0.3)	513 (55)
non-*Trichodesmium* OTUs only	Puff	5.6 (0.6)	250 (66)
	Tuft	4.4 (0.6)	212 (57)
	Mixed	5.8 (0.3)	269 (38)
	SW	5 (0.3)	426 (27)

*Estimates are derived from partial 16S rRNA gene sequences using all OTUs (7011 sequences per sample) and OTUs excluding Trichodesmium sequences (3128 sequences per sample). Data are presented as averages within sample type (n = 8 puff, 5 tuft, 4 mixed morphology, and 4 25 m bulk seawater (SW) samples), with standard deviations in parentheses*.

### Diazotroph diversity via *nifH* amplicons

**Figure 5:**
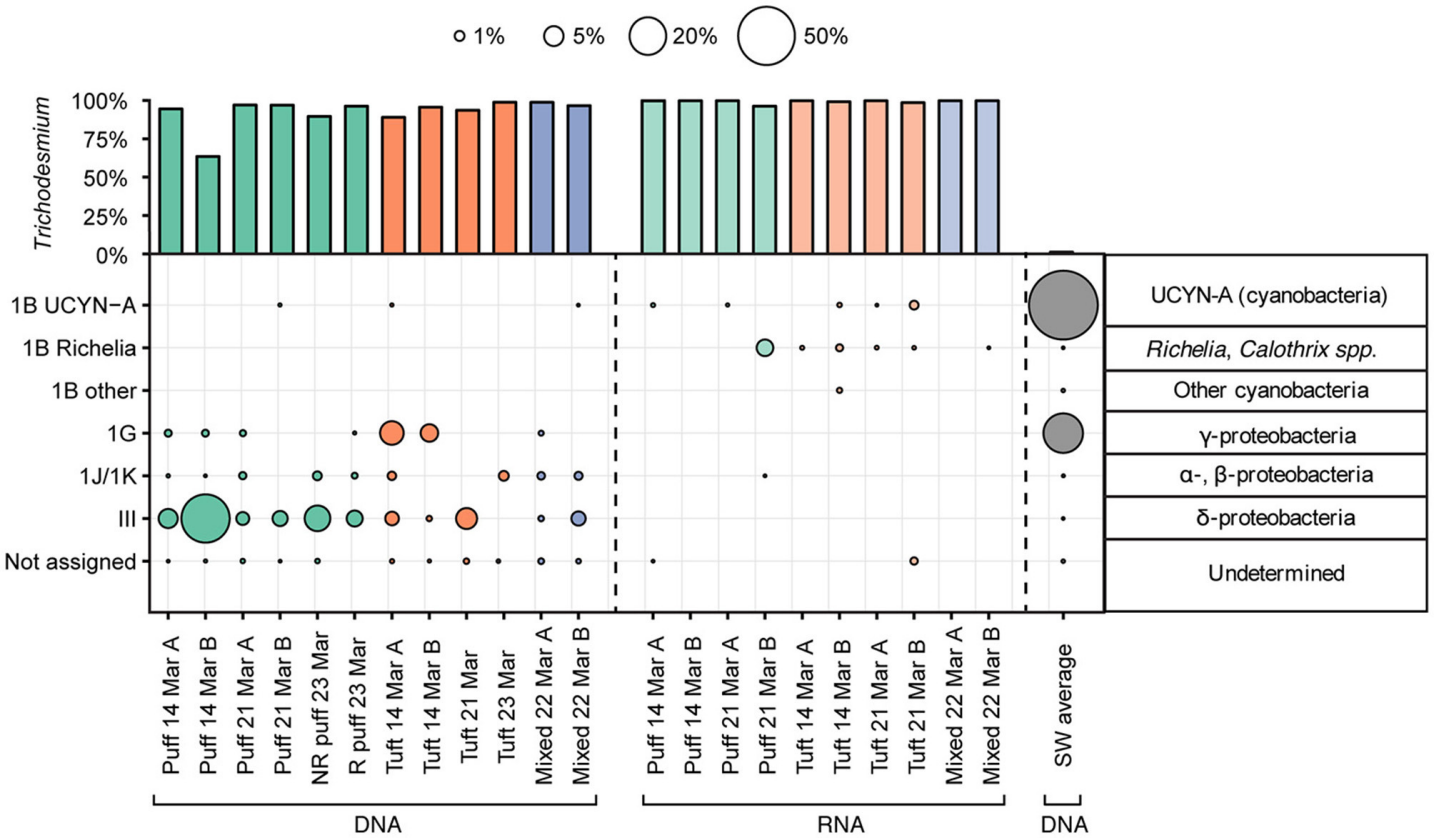
Percentages of *nifH* gene (left, right) and transcript (center) sequences assigned to *nifH* cluster groups. Percentages of *Trichodesmium* sequences are displayed in the upper bar plot; sequences assigned to other taxa are displayed in the lower area plot. Representative taxa from canonical *nifH* clusters (Zehr et al., 2003) are shown to the right. Color indicates the sample type/morphology [green, red, blue, and gray for puff colonies, tuft colonies, mixed colonies, and bulk 25 m seawater (SW), respectively]. R denotes radial and NR denotes non-radial puff morphologies (see Figure 1).

We sequenced partial *nifH* genes and transcripts from *Trichodesmium* colonies, and from surface seawater samples for comparison, to test for the presence and transcriptional activities of non-*Trichodesmium* diazotrophs associated with the colonies. While sequences belonging to *Trichodesmium* dominated the *nifH* dataset, we also recovered non-*Trichodesmium nifH* genes and transcripts (Figure 5). In the DNA samples, *Trichodesmium* represented 64–99% of *nifH* sequences, with an average of 7% of sequences corresponding to non-*Trichodesmium* diazotrophs. Most non-*Trichodesmium nifH* DNA sequences were classified as *nifH* Cluster III, a group that includes anaerobic microorganisms, such as *Desulfovibrio* and *Clostridium* (Zehr et al., 2003). Non-*Trichodesmium* groups other than Cluster III represented 1.5% of *nifH* gene sequences, and included previously identified *nifH* groups, such as 1G (presumed Gammaproteobacteria), 1J/1K (presumed Alpha- and Betaproteobacteria), and a very small percentage of sequences belonging to the cyanobacterium UCYN-A. The non-*Trichodesmium* diazotrophs associated with the colonies were distinct from diazotrophic taxa in the surrounding seawater, where *nifH* gene sequences were dominated by UCYN-A and presumed Gammaproteobacteria and contained <0.01% *nifH* Cluster III.

A much smaller fraction of *nifH* transcript sequences belonged to non-*Trichodesmium* diazotrophs (Figure 5). Sequences phylogenetically related to the *nifH* Cluster III, 1G, and 1J/1K, which constituted a modest proportion of *nifH* gene sequences, were conspicuously absent from the *nifH* transcript sequences. The small fraction of non-*Trichodesmium nifH* transcripts (0–3.5%) belonged to Cyanobacteria, predominantly cyanobacterium UCYN-A and *Richelia/Calothrix* (with the exception of one sample containing 0.01% 1J/1K, presumed Alpha- and Betaproteobacteria).

The *nifH* DNA and RNA sequences show that *Trichodesmium* puff and tuft colonies harbored different communities of non-*Trichodesmium* diazotrophs (Figure 5). Puff colonies harbored a larger fraction of Cluster III (average 9.6% of *nifH* gene sequences) than tuft colonies (average 2.1% of *nifH* DNA sequences), while tuft colonies harbored a larger fraction of 1G (presumed Gammaproteobacteria, average 2.9% of *nifH* gene sequences) than puff colonies (average 0.2% of *nifH* gene sequences). Additionally, *nifH* transcripts from puff and tuft colony morphologies included different phylotypes of heterocystous Cyanobacteria (*Richelia*/*Calothrix*). One puff RNA sample contained transcripts derived from the *Calothrix* SC01/HET-3 group (Foster and Zehr, 2006; Foster et al., 2010). No tuft samples contained *Calothrix* SC01 sequences, but all 4 tuft RNA samples contained transcripts derived from the *Richelia*/HET-1 group (Church et al., 2005b). Neither heterocystous phylotype matched qPCR primer sets developed by Momper et al. (2015) to target the heterocystous cyanobiont hetDA (≥4 mismatches with forward primer for both phylotypes; reverse primer was out of our sequencing region).

### Metagenomic taxonomy and functional potential

We sequenced metagenomes from two *Trichodesmium* puff samples collected on 23 Mar 2014 (“radial puff” and “non-radial puff”) and assembled and annotated these sequences along with sequences from a publically available Stn. ALOHA surface seawater metagenome collected in July 2015. Colony metagenomes were dominated by bacteria (>99% of total counts), with ~70% of counts assigned to Cyanobacteria (Table 4). Cyanobacteria accounted for an average of 57 and 67% of counts from a set of 29 single-copy genes (Table S4) from radial and non-radial puff colonies, respectively. Thus, assuming one copy of each of these genes per genome (Nayfach and Pollard, 2015), equal levels of ploidy among taxa, and that the majority of cyanobacterial counts belong to *Trichodesmium*, both total metagenome and single-copy gene counts produce conservative estimates of ~1 epibiont cell for every 1–3 *Trichodesmium* cells within colonies. Less than 1% of *Trichodesmium* colony counts were assigned to Eukarya, Archaea, or viruses, compared to 3.6% of surface seawater counts (Table 4). Eukaryotes represented 0.5 and 0.3% of non-radial and radial puff colony counts, respectfully, with dominant groups including green algae (Streptophyta and Chlorophyta), chordates, heterotrophic flagellates (Choanoflagellida), arthropods, diatoms, ciliates, and fungi (Table 4, Table S5). The relative abundances of bacterial taxa mirrored trends observed in the 16S rRNA gene dataset, with the majority of colony sequences belonging to Cyanobacteria (primarily *Trichodesmium*), Alphaproteobacteria, Bacteroidetes, and Gammaproteobacteria (Table 4, Figure 4).

**Table 4:** Taxonomic assignments from three metagenome samples.

	***Trichodesmium* non-radial puff colonies (%)**	***Trichodesmium* radial puff colonies (%)**	**Stn. ALOHA surface seawater (%)**
Cyanobacteria	75.9	65.5	31.8
α-proteobacteria	11.1	12.5	41.6
γ-proteobacteria	3.2	5.3	11.2
δ-proteobacteria	1.2	0.9	0.8
β-proteobacteria	0.5	1.1	0.7
Bacteroidetes	4.1	8.6	5.4
Firmicutes	1.1	1.3	1.3
Planctomycetes	0.8	1.9	0.3
Actinobacteria	0.5	0.6	1.0
Verrucomicrobia	0.1	0.5	0.6
Chloroflexi	0.1	0.2	0.1
Eukaryota	0.5	0.3	2.6
Archaea	0.1	0.2	0.5
Viruses	0.1	0.1	0.5
Other bacteria	0.7	1.0	1.6

*Values denote percentages of length-corrected reads mapped to annotated assemblies. Trichodesmium and surface seawater metagenomes are derived from samples collected 23 Mar 2014 and 30 July 2015, respectively*.

Metagenome counts were annotated to KO and normalized to GPM in order to compare the relative abundance of genes and pathways among samples. However, ~75% of assembled contigs from *Trichodesmium* colonies failed KO annotation, far exceeding the ~25% of failed contig annotations observed in the surface seawater sample (Table 2). Furthermore, of the sequences that were successfully mapped to annotated contigs, *Trichodesmium* samples contained a larger fraction of KO with unknown function than the surface seawater sample (Table 2). This resulted in smaller GPM values from *Trichodesmium* metagenomes than the surface seawater metagenome for most KEGG gene categories (Figure S3). Hence, we chose to use a normalization of counts per million mapped to a KO of known function (GPMK) in order to compare the functional potential of *Trichodesmium* colonies and surface seawater.

**Figure 6:**
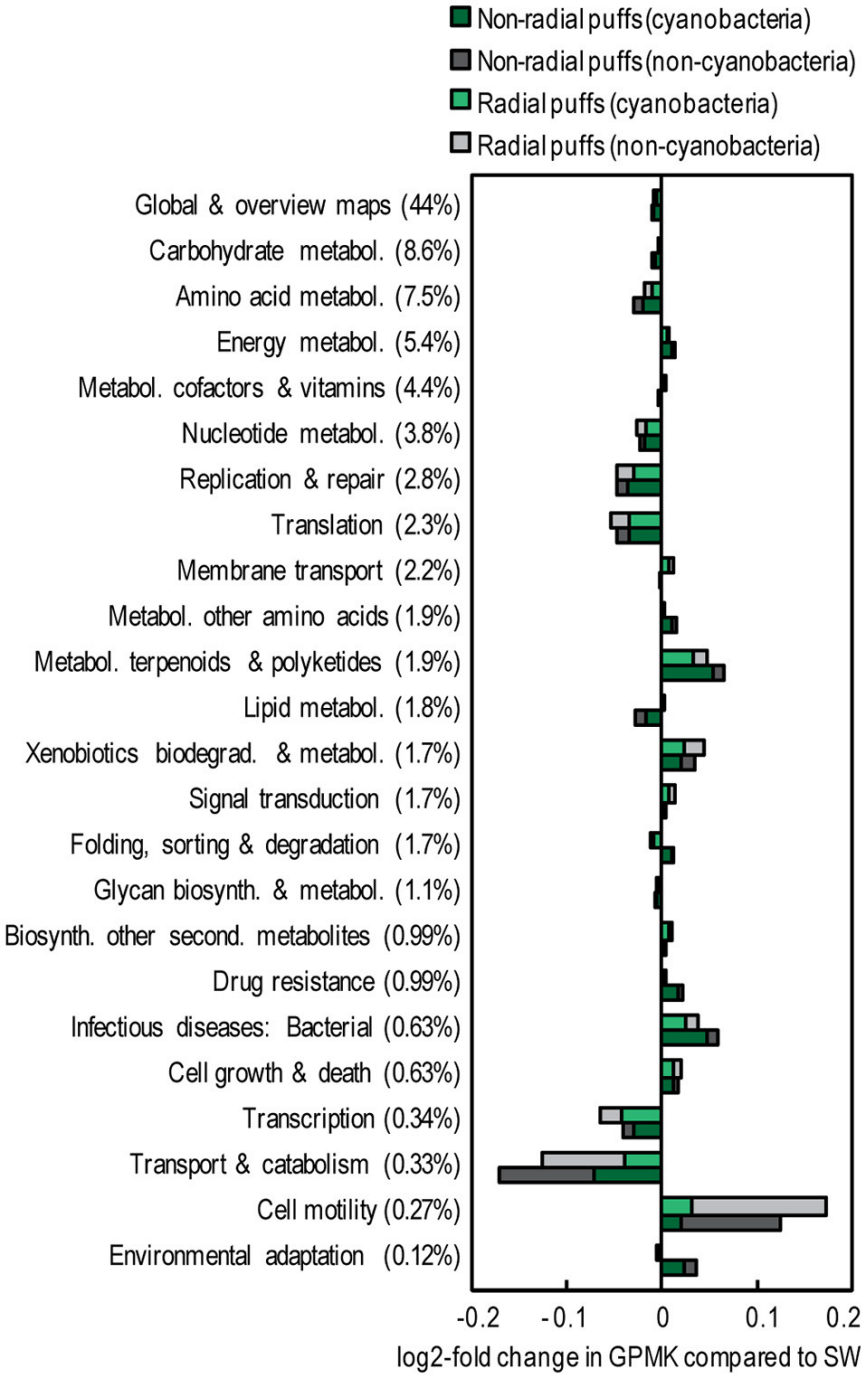
Relative abundance of KEGG gene groups in *Trichodesmium* colony samples (collected 23 Mar 2014) compared to a surface seawater sample from Stn. ALOHA (collected 30 July 2015). The percentages of total counts within each gene group are provided in parentheses. Colors represent sample type and taxonomic assignment. Pathways involved with organ systems, human disease, and/or representing <0.1% of total GPMK were excluded. Pathways displayed represent >97% of total GPMK.

The gene contents of the *Trichodesmium* colony samples were distinct from those observed in the near-surface seawater. Colonies contained ~40% fewer single-copy GPM than the seawater samples (both in Cyanobacteria and non-Cyanobacteria fractions, Table 2, Table S4), suggesting larger average genome sizes for *Trichodesmium* and epibiont cells. Summing KOs from KEGG gene groups revealed broad functional differences between colonies and surface seawater (Figure 6). Seawater samples were relatively enriched in KEGG groups including nucleotide and amino acid metabolism, transcription, translation, and replication and repair, while the colony samples were relatively enriched in energy metabolism, metabolism of terpenoids and polyketides, and cell motility.

**Figure 7:**
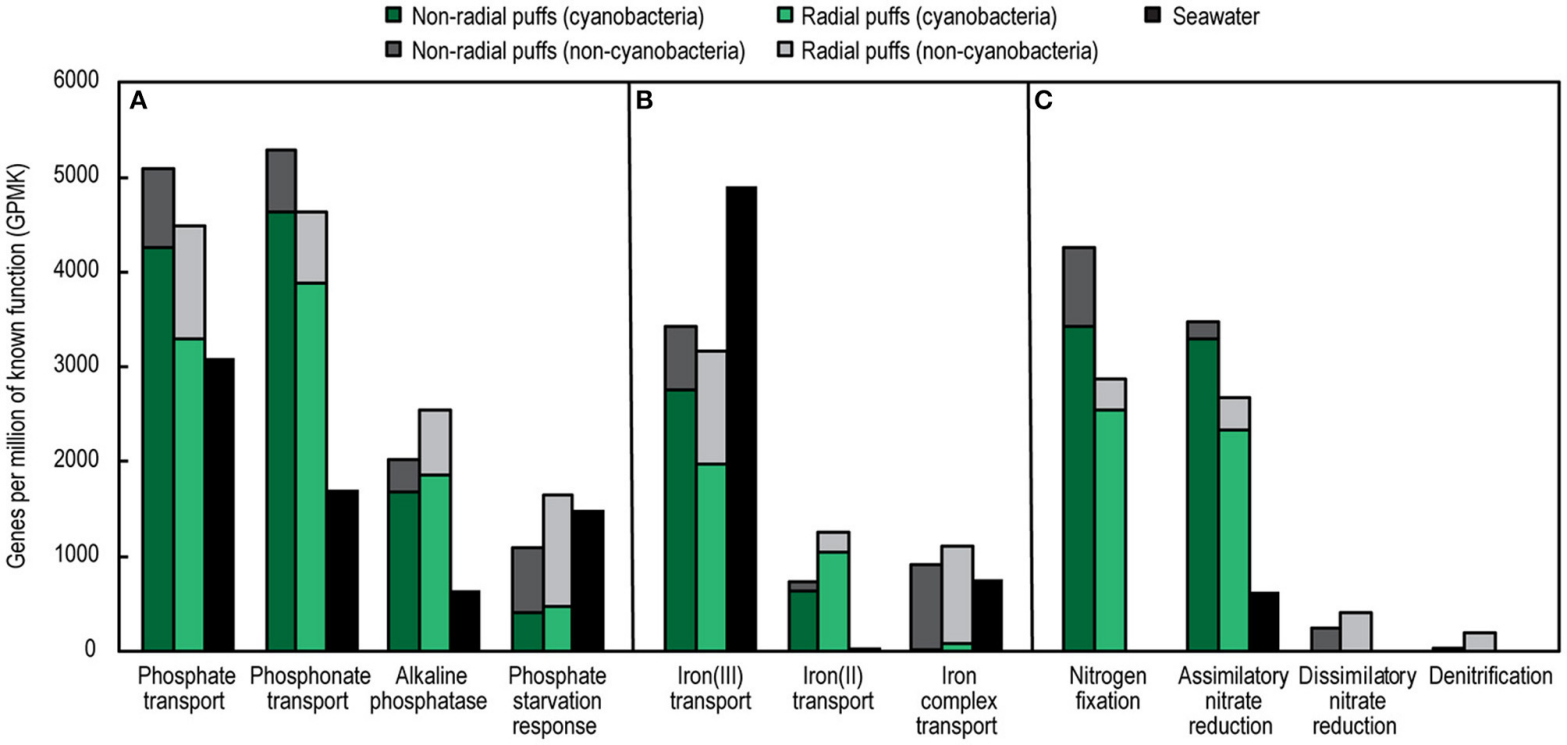
Abundances of select genes and pathways involved in phosphorus **(A)**, iron **(B)**, and nitrogen **(C)** cycling from *Trichodesmium* colony and surface seawater metagenomes. Colors represent sample type and taxonomic assignment. See Table S6 for a list of KO included in each pathway.

*Trichodesmium* colony and surface seawater metagenomes also differed in the abundances of specific genes and pathways involved in nutrient cycling (Figure 7). Colonies were enriched in genes encoding alkaline phosphatase and transporters for phosphate, phosphonates, and Fe(II), but depleted in Fe(III) transporter genes, compared to seawater. There were similar abundances of phosphate starvation response and Fe complex (siderophore) transport genes in colonies and seawater; however, the majority of these genes in the colonies belonged to non-Cyanobacteria (epibionts), which only represented ~30% of total colony metagenome counts. Thus, phosphate starvation response and Fe complex transport genes were enriched in epibionts compared to the surrounding plankton.

*Trichodesmium* colonies were also enriched in N cycling genes. Compared to seawater, the colony metagenomes contained higher total N metabolism gene abundances (34 and 46% higher abundances in radial and non-radial puffs, respectively, Table S6), and were strongly enriched in genes involved in N transformation pathways (Figure 7). Nitrogenase genes were ~2,000X more abundant in colonies than seawater, and included a large fraction assigned to non-Cyanobacteria (11 and 20% of nitrogenase genes in radial and non-radial puff colonies, respectively). Assimilatory nitrate reduction genes were present in both colony and seawater samples, but were ~5X more abundant in colonies, where the majority of genes corresponded to Cyanobacteria. Dissimilatory nitrate reduction and denitrification genes were absent in seawater samples but present in both colony samples; genes in these pathways were nearly exclusively assigned to non-Cyanobacteria (Figure 7). Genes involved in nitrification pathways were not observed in colony or seawater metagenomes.

## Discussion

Several decades of research have documented the presence of bacterial and eukaryotic epibionts inhabiting *Trichodesmium* colonies (Borstad and Borstad, 1977; Siddiqui et al., 1992; Rouco et al., 2016), but the taxonomic composition and functional potential of these associated communities are not well-understood. Here, we used a variety of molecular tools to probe the diversity of *Trichodesmium* and associated epibionts in colonies from the NPSG. We found that the colonies were dominated by a single clade of *Trichodesmium*, but harbored a diverse community of associated microorganisms. These microbial assemblages were distinct from the surrounding seawater, differed by colony morphology, and included bacteria with a known preference for surface attachment, as well as putative anaerobic diazotrophs. Colony metagenomes contain genes and pathways not present in *Trichodesmium* genomes, including siderophore transport and denitrification genes, which likely affects the biogeochemical functioning of *Trichodesmium* colonies.

### *Trichodesmium* species diversity

The abundance and distribution of *Trichodesmium* have been studied extensively, but most work has focused on *Trichodesmium* at the genus-level, using techniques including microscopy, video plankton recording, and satellite imaging (e.g., Dugdale, 1961; Subramaniam et al., 2001; Davis and McGillicuddy, 2006). In the laboratory, *Trichodesmium* isolates have been phylogenetically classified into four major clades (based on the hetR and ITS genes), with the majority of isolates falling into Clade I (e.g., *T. thiebautii*) and Clade III (e.g., *T. erythraeum*) (Orcutt et al., 2002; Hynes et al., 2012), but the geographical distributions of these clades in field populations has only begun to be investigated. Our finding of Clade I dominance is in agreement with recent surveys in the N. Pacific, N. Atlantic, and S. Pacific (Hmelo et al., 2012; Gradoville et al., 2014; Rouco et al., 2014, 2016), which all observed the majority of *Trichodesmium* sequences belonging to Clade I. However, most physiological studies of *Trichodesmium* use the cultivated Clade III laboratory isolate *T. erythraeum* IMS101. Isolates from Clade I and Clade III appear to respond differently to environmental stimuli; for example, elevating *p*CO_2_ enhances rates of N_2_ and C fixation by Clade III isolates IMS101 and GBRTRLI101 but not by the Clade I isolate H9-4 (Hutchins et al., 2007, 2013). While more work is needed to resolve the spatial and temporal variability of *Trichodesmium* species biogeography, current evidence suggests that at a global scale *Trichodesmium* Clade I may be more abundant than Clade III. Hence, modeling studies using the response of isolate IMS101 to predict the *p*CO_2_ response of natural *Trichodesmium* populations should be viewed with caution. In addition, our findings agree with previous reports that colony morphology is not an accurate proxy for *Trichodesmium* clade (Hynes et al., 2012), as both puff and tuft colony samples were composed of >99% Clade I (Figure 2).

### Diversity of associated microbiome

Our samples contained diverse bacterial and eukaryotic taxa associated with *Trichodesmium* colonies. While relative abundances derived from metagenomic and 16S rRNA gene sequences can be biased by taxa-specific genome sizes and copy numbers of genes and genomes (e.g., Sargent et al., 2016), respectively, the large fractions of non-*Trichodesmium* sequences in both datasets suggest that epibionts are numerically abundant within the colonies. Colony metagenome sequences were dominated by bacteria, but we also observed sequences from viruses, Archaea, and many eukaryotic taxa previously observed associated with *Trichodesmium* colonies (Borstad and Borstad, 1977; Sheridan et al., 2002). Bacterial species richness within colonies was ~10-fold higher than the richness previously assessed for Atlantic colonies using clone libraries (Hmelo et al., 2012), and approximately half of the richness in surrounding seawater, reaffirming that colonies harbor a diverse epibiont community (Sheridan et al., 2002; Rouco et al., 2016).

The taxonomic composition of colony epibionts was distinct from that of the surrounding bacterioplankton. The warm, oligotrophic waters of the NPSG are known to be dominated by the Cyanobacteria *Prochlorococcus* (Campbell et al., 1994) and photo- and chemoheterotrophs including SAR11 and Rhodobacteraceae (DeLong et al., 2006). Indeed, the most abundant taxa in our near-surface seawater samples were those clustering among *Prochlorococcus, Synechococcus*, and the small photoheterotroph Actinomarina (SAR11 represented only 3.2% of seawater sequences, likely due to a known bias in the 16S rRNA gene primer set used, Apprill et al., 2015); however, these taxa were all conspicuously absent from *Trichodesmium* colony samples. The relative absence of typical oligotrophic bacteria with streamlined genomes in colonies could be due to elevated nutrient concentrations favoring copiotrophic taxa (Lauro et al., 2009; Giovannoni et al., 2014), and is consistent with previous observations of large marine particle size classes being enriched with copiotrophic bacterial genes (Allen et al., 2013). Instead, colony epibionts were dominated by Bacteroidetes, Alphaproteobacteria, and Gammaproteobacteria, which is consistent with previous 16S rRNA gene surveys of microbial communities associated with *Trichodesmium* (Hmelo et al., 2012; Rouco et al., 2016). Several dominant epibiont taxa have been previously observed associated with marine particulates, including the Bacteroidetes classes Cytophagia and Flavobacteriia (DeLong et al., 1993; Crump et al., 1999; Bryant et al., 2016), Alteromonadales (Fontanez et al., 2015), and Planctomycetes (DeLong et al., 1993). Though epibiont communities had several abundant taxa in common with the surrounding seawater at the order-level (e.g., Rhodobacterales, Rhodospirillales, and Oceanospirillales), there were few commonalities with surface seawater phylotypes at the 97% OTU-level. The distinct community structure and lower species richness of epibionts compared to surrounding bacterioplankton, and the commonalities between epibiont taxa from our samples and previous *Trichodesmium* studies (Hmelo et al., 2012; Rouco et al., 2016) together suggest that *Trichodesmium* colonies provide a niche favoring select bacterial taxa.

In addition, we observed distinct epibiont communities associated with puff and tuft colonies, in agreement with Rouco et al. (2016), as well as evidence that certain bacterial species may consistently associate with specific morphotypes. Tuft colonies contained a larger fraction of *Trichodesmium* 16S rRNA gene sequences than puff colonies, possibly due to less colonizable surface area in this morphotype, which likely drives the lower diversity values observed for tufts (Table 3). This finding contrasts with the microscopic observations of Sheridan et al. (2002), who reported tuft colonies harboring higher bacterial densities than puff colonies. Furthermore, the epibiont composition differed between the two morphologies, both in terms of phyla-level taxonomy (e.g., puffs contained more Bacteroidetes and tufts contained more non-*Trichodesmium* Cyanobacteria, Figure 4) and, even more strikingly, in the relative abundance of specific phylotypes (Figure S2). For example, a phylotype clustering among the filamentous Cyanobacteria *Limnothrix* represented 11.7% of non-*Trichodesmium* tuft sequences but only 0.1% of non-*Trichodesmium* puff sequences. Filamentous Cyanobacteria have been observed in close association with *Trichodesmium* filaments from tuft colonies (e.g., Paerl et al., 1989a; Siddiqui et al., 1992; Hewson et al., 2009), and *Limnothrix*-like sequences represented 31% of 16S rRNA gene clone library sequences in tuft (but not puff) colonies from the N. Atlantic (Hmelo et al., 2012). Thus, this *Limnothrix* phylotype may be a common associate of *Trichodesmium* tufts. Likewise, *Microscilla* represented 7.7% of tufts but only 0.01% of non-*Trichodesmium* puff sequences, and this genus has been previously recovered from *Trichodesmium* tufts in the N. Pacific, N. Atlantic, and Caribbean Sea (Janson et al., 1999; Rouco et al., 2016). Puff colonies also contained abundant phylotypes which were relatively absent from tufts, including Alphaproteobacteria and Bacteroidetes phylotypes, the cyanobacterium *Rivularia*, and a *Marinicella* phylotype which shares 100% nucleotide sequence identity to a sequence previously recovered from *Trichodesmium* colonies (accession GU726121). It is remarkable that so many phylotypes had significantly different relative abundances between the two morphotypes (Figure S2), and also that many of the most abundant genera from our samples have also been dominant in previous surveys of *Trichodesmium* epibionts (Hmelo et al., 2012; Rouco et al., 2016). Since the species composition of *Trichodesmium* did not vary by colony morphology, physical (i.e., filament compactness, colonizable surface area) and/or chemical properties of puff and tuft colonies likely drive the observed differences in epibiont community structure.

### Colony-associated diazotrophs

There have been several reports of cyanobacterial and heterotrophic diazotrophs associated with *Trichodesmium* colonies (Paerl et al., 1989b; Gradoville et al., 2014; Momper et al., 2015), but the community composition and metabolic activity of these organisms have been largely unexplored. Here, we used high-throughput sequencing of partial *nifH* genes and transcripts to explore the diversity of colony-associated diazotrophs. We observed non-*Trichodesmium nifH* genes (including genes from putative heterotrophs) in all *Trichodesmium* DNA samples, representing 1–35% of the colony *nifH* sequences (Figure 5).

The ecological importance of non-cyanobacterial marine diazotrophs is a current enigma in N_2_ fixation research: non-cyanobacterial *nifH* genes have been recovered from numerous marine environments (Bombar et al., 2016), but rates of N_2_ fixation in marine environments dominated by non-cyanobacterial diazotrophs are often low or undetectable (e.g., Knapp et al., 2016; Gradoville et al., 2017). Here, we found robust evidence that *Trichodesmium* colonies comprise yet another habitat for these seemingly cosmopolitan organisms. The majority of our non-*Trichodesmium nifH* gene sequences phylogenetically grouped among Cluster III *nifH* genes, which includes diverse anaerobic microorganisms (Zehr et al., 2003). The possibility of anaerobic bacteria inhabiting *Trichodesmium* colonies appears plausible since colonies have been reported to contain anoxic microzones (Paerl and Bebout, 1988); indeed, we also found denitrification and Fe(II) transporter genes enriched in colony metagenomes (see *Functional potential within Trichodesmium colonies*). However, *nifH* Cluster III contains diverse lineages (Zehr et al., 2003), and the physiology and ecology of these organisms are not well-understood. In our study, the three most abundant Cluster III OTUs each share <85% nucleotide identity with any cultured representative in the BLASTn database. One of these OTUs matches a qPCR primer/probe set designed by Church et al. (2005a) to quantify a specific group of Cluster III *nifH* sequence-types in the NPSG, while all three OTUs share >99% nucleotide identity with sequences previously obtained from *Trichodesmium* colonies at Stn. ALOHA (Gradoville et al., 2014). Such results suggest that *Trichodesmium* colonies may selectively harbor members of the Cluster III *nifH* phylotypes, including organisms not currently captured by existing Cluster III qPCR primers and probes (Church et al., 2005a).

It is interesting to note that both Cluster III and the 1J/1K (presumed Alpha- and Betaproteobacteria) group had higher relative abundances in our *Trichodesmium* colony samples than in the surrounding seawater, where *nifH* sequences were dominated by the unicellular cyanobacterium UCYN-A, the Gammaproteobacterial *nifH* group 1G, and other Cyanobacteria including *Trichodesmium* (Figure 5). This suggests that *Trichodesmium* colonies may represent a niche for Cluster III and 1J/1K diazotrophs. It is possible that the relative enrichment of these groups in *Trichodesmium* colonies could reflect a preference for marine particulates—for example, Bryant et al. (2016) observed marine plastic particles to be enriched in *nifH* genes—rather than a unique property of the colonies themselves. Marine particles may be favorable environments for heterotrophic diazotrophs (Bombar et al., 2016), especially putative anaerobic Cluster III taxa, which could inhabit anoxic microzones of particles (Benavides et al., 2015). Future research is needed to determine whether *Trichodesmium* colonies represent an important niche for *nifH* Cluster III diazotrophs in the NPSG and other oceanic regions.

Though *Trichodesmium nifH* amplicons included genes belonging to non-Cyanobacteria, the absence of non-cyanobacterial *nifH* transcripts suggests that these taxa were not actively fixing N_2_ at the time of sampling (Figure 5). However, we did observe non-*Trichodesmium* cyanobacterial *nifH* transcripts, mostly belonging to two OTUs in the heterocystous *Calothrix/Richelia* group. One of the *Calothrix*/*Richelia* OTUs matched primer/probe sets for group HET-1 (Church et al., 2005b), and was present in all tuft RNA samples, while another *Calothrix*/*Richelia* OTU, present in one puff RNA sample, matched primer/probe sets for the SC01/HET-3 group (Foster and Zehr, 2006; Foster et al., 2010). This suggests that there may be morphotype-specific associations between heterocystous Cyanobacteria and *Trichodesmium* other than the cohabitation described by Momper et al. (2015). While *Calothrix*/*Richelia* sequences were absent in the *nifH* DNA dataset (likely due to poor amplification of this group by the *nifH* primers used; Turk-Kubo et al., 2015), we did see evidence of heterocystous Cyanobacteria inhabiting colonies in the 16S rRNA datset (*Rivularia* and a small number of *Richelia* sequences; Figure S2, Table S3). Furthermore, the presence of *Calothrix*/*Richelia nifH* transcripts indicates high cell-specific transcription rates by this group. Our observation of higher *nifH* transcription levels in cyanobacterial diazotrophs than non-cyanobacterial diazotrophs is consistent with previous *nifH* gene expression surveys using bulk seawater from Stn. ALOHA (Church et al., 2005b).

### Functional potential within *Trichodesmium* colonies

Our metagenomic data suggest that *Trichodesmium* epibionts may benefit from a colony-associated lifestyle and influence nutrient cycling within colonies. Epibionts appeared to possess larger average genome sizes than bulk plankton, suggesting non-streamlined genomes, consistent with the relative absence of oligotrophic taxa (*Prochlorococcus, Actinomarina*, etc.) observed in colonies. Furthermore, epibionts were depleted in genes involved in replication and basic metabolic functioning relative to seawater metagenomes, again consistent with a lack of streamlined genomes (Giovannoni et al., 2014). Instead, colony samples were enriched in genes involved in motility, which could be useful in a colony-associated lifestyle, and in metabolic pathways not present in the seawater metagenome (Figures 6, 7, Table S6). Additionally, a large fraction of *Trichodesmium* colony contigs failed annotation. This could be due to the large fraction of non-coding DNA in the *Trichodesmium* genome (Walworth et al., 2015), but could also arise from a larger fraction of uncultivated microorganisms in the *Trichodesmium* microbiome than in the surrounding seawater. Likewise, colony samples contained a larger fraction of genes with unknown function than seawater samples. Our findings of enrichments in copiotrophic taxa, motility genes, and genes of unknown function within *Trichodesmium* colonies have also been observed in particle-attached marine microbial communities (Simon et al., 2014).

The NPSG is a chronically oligotrophic system, with production rates limited by the availability of N (Karl et al., 1997) and sometimes P (Karl et al., 1995). Since diazotrophs such as *Trichodesmium* circumvent N limitation through N_2_ fixation, their growth and N_2_ fixation rates are typically limited by the availability of P and/or Fe (as well as light and temperature, Luo et al., 2014). Hence, there is considerable interest in understanding the mechanisms of P and Fe acquisition by *Trichodesmium*. We observed enrichments in alkaline phosphatase, phosphate transport, and phosphonate transport genes in colonies, which agrees with previous demonstrations of efficient organic phosphorus scavenging and utilization by *Trichodesmium* (Dyhrman et al., 2006). Furthermore, colony epibionts contained genes encoding the synthase for acyl homoserine lactoses (Table S6), quorum sensing molecules which have been shown to stimulate alkaline phosphatase activity by *Trichodesmium* cells in culture (Van Mooy et al., 2011). We also found that phosphate starvation response genes were enriched in epibionts, which could reflect P-limitation due to the release of inorganic and organic N compounds by *Trichodesmium* cells (Capone et al., 1994; Mulholland et al., 2004).

The genes involved in Fe transport also differed between colony and seawater metagenomes. Fe(II) transporters were enriched in colony samples, consistent with previous observations of these genes in *Trichodesmium* isolates (Chappell and Webb, 2010), but were nearly absent in the seawater metagenome. In well-oxygenated seawater, most Fe exists as Fe(III), hence our observation of epibionts enriched in Fe(II) transport genes suggests that low-oxygen microzones within colonies could result in reduction of Fe(III). Additionally, we found low abundances of cyanobacterial siderophore transport genes, reflecting the inability of *Trichodesmium* to use highly chelated Fe sources (Chappell and Webb, 2010), but these genes were enriched in non-cyanobacterial epibionts. Our observations of abundant P and Fe acquisition genes in *Trichodesmium* and epibionts could reflect competition for these resources in the colony community. However, we also found metagenomic evidence for previously described potential mutualisms, as epibionts could facilitate *Trichodesmium* nutrient uptake through quorum sensing (Van Mooy et al., 2011) and siderophore production (Roe et al., 2012).

Finally, we observed the genetic capacity for denitrification within *Trichodesmium* colonies. Both colony samples contained all necessary genes for the denitrification and dissimilatory nitrate reduction pathways, while no genes from either pathway were observed in the seawater sample. Furthermore, 16S OTU 18, comprising 9.9% of non-*Trichodesmium* 16S rRNA gene sequences from tuft colonies, was classified as the denitrifier *Nisaea* sp. These results agree with Wyman et al. (2013), who reported *nosZ* amplicons isolated from *Trichodesmium* colonies in the Arabian Sea. Denitrification within the colonies would be biogeochemically significant, producing a tight spatial coupling between N_2_ fixation and denitrification and reducing apparent colony N_2_ fixation rates. However, denitrification requires nitrate, and we did not observe any nitrification genes within colonies (Table S6), although it is possible that nitrate could be supplied through diurnal migration to deeper nitrate-rich waters (Walsby, 1978). Furthermore, denitrification is an anaerobic process, and while early reports indicated that colonies could contain anoxic zones (Paerl and Bebout, 1988), more recent work has found no evidence for this (Eichner et al., 2017). Thus, it is possible that the presence of denitrification genes does not indicate active denitrification within colonies, but rather reflects the diverse gene repertoire of copiotrophic epibionts.

## Conclusions

Our multifaceted high-throughput sequencing approach enabled a detailed view of the *Trichodesmium* colony microbiome. While the species composition of *Trichodesmium* was dominated by a single clade and uniform in all of our samples, the community structure of bacterial epibionts differed between puff and tuft colony morphologies, suggesting that differences in biogeochemical rates among colony morphologies may be driven by processes carried out by the associated microbiome. Epibionts appear copiotrophic, with the genetic capacity to influence colony nutrient cycling. Additionally, we found that colonies contained active cyanobacterial diazotrophs and presumed heterotrophic and anaerobic diazotrophs, suggesting that *Trichodesmium* colonies harbor a unique microbial community with the potential to influence rate processes classically attributed to *Trichodesmium* spp.

## Author contributions

This study was conceived by MG. Data were collected by MG and analyzed by MG, BC, MC, RL, and AW. MG wrote the first draft of the manuscript. All authors contributed substantial revisions through the drafting process and approved the final submitted manuscript.

### Conflict of interest statement

The authors declare that the research was conducted in the absence of any commercial or financial relationships that could be construed as a potential conflict of interest.
