# Corrigendum: Microbiome of *Trichodesmium* Colonies from the North Pacific Subtropical Gyre

**DOI:** 10.3389/fmicb.2017.01780

**Published:** 2017-09-19

**Authors:** Mary R. Gradoville, Byron C. Crump, Ricardo M. Letelier, Matthew J. Church, Angelicque E. White

**Affiliations:** ^1^College of Earth, Ocean and Atmospheric Sciences, Oregon State University Corvallis, OR, United States; ^2^Flathead Lake Biological Station, University of Montana MT, United States

**Keywords:** *Trichodesmium*, marine microbiome, *nifH* diversity, heterotrophic marine diazotrophs, metagenomics, 16S rRNA, nitrogen fixation

In the original article, there were two errors. First, an incorrect NCBI accession number was provided. A correction has been made to Methods, Bioinformatic Analyses, Paragraph 4:

“All raw sequences are available from NCBI (accession SRP095769). Assemblies and annotation data are available from IMG/M ER (http://img.jgi.doe.gov/mer; Taxon OIDs 3300009572, 3300009536, and 3300010936).”

Additionally, in the original article, the two primer sets used for nested *nifH* PCR were listed in the incorrect order and included an incorrect reference. A correction has been made to Methods, Nucleic Acid Extraction, Amplification, and Sequencing, Paragraph 3:

“The *nifH* gene was amplified using nested degenerate *nifH* primers (Zehr and McReynolds, 1989; Zani et al., 2000). The first round contained 1X PCR buffer, 0.1U Platinum High Fidelity Taq polymerase (Invitrogen), 200 μmol L^−1^ dNTPs, 3% BSA, 4 mmol L^−1^ Mg^2+^, 1 μL DNA or cDNA, and 1 μmol L^−1^ nifH3 and nifH4 primers (Zani et al., 2000). Reaction conditions were: 94°C for 7 min, followed by 30 cycles of 94°C for 1 min, 57°C for 1 min, and 72°C for 1 min, and a final 72°C extension for 7 min. The second round of *nifH* PCR used the same components and thermocycling conditions as the first round, except the DNA extract was replaced with 1 μL of the amplified product generated during the first round PCR reaction, and custom primers were used, consisting of gene-specific sites (nifH1 and nifH2), dual- indexed barcodes, Illumina linkers, and a sequencing primer binding region, similar to those described by Kozich et al. (2013; Table S1). PCR negative controls and filter blank samples were included in PCR reactions.”

Finally, there was a mistake in the legend for Supplementary Table 1 as published. This table listed the incorrect *nifH* primer names. The correct legend appears below.

Table S1: Dual-index barcoded, forward and reverse *nifH* primers (5′ -> 3′) used in this study. Sample barcodes are shown in bold. Forward and reverse *nifH* PCR primers are indicated by nifH1 (TGYGAYCCNAARGCNGA) and nifH2 (ADNGCCATCATYTCNCC; note the misprint of this primer in the original manuscript by Zani et al., 2000). NNNN indicate Illumina linker regions: AATGATACGGCGACCACCGAGATCTACAC (forward) and CAAGCAGAAGACGGCATACGAGAT (reverse). Blue text indicates the binding site for sequencing primers, which were designed to optimize melting temperature during sequencing, as described by Kozich et al. (2013).

The authors apologize for these errors and state that they do not change the scientific conclusions of the article in any way.

## Conflict of interest statement

The authors declare that the research was conducted in the absence of any commercial or financial relationships that could be construed as a potential conflict of interest.
