# Supplementary material for: Microbiome of Trichodesmium Colonies from the North Pacific Subtropical Gyre
Source: Front Microbiol. 2017 Jul 6;8:1122. doi: 10.3389/fmicb.2017.01122 (PMC5498550; doi:10.3389/fmicb.2017.01122)
Supplement: Supplementary file 7 [file Image1.PDF]

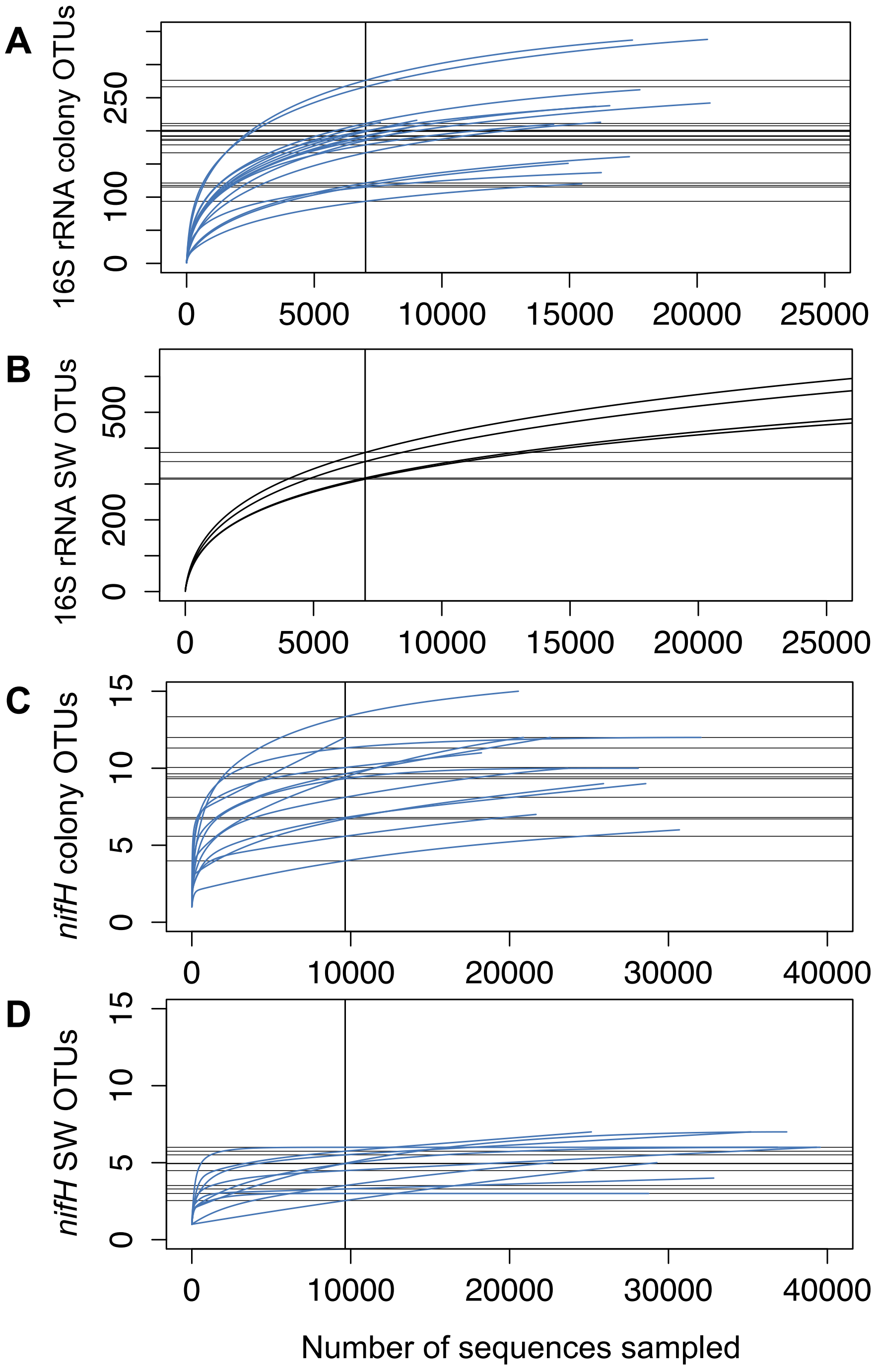

Figure S1: Rarefaction curves for *Trichodesmium* colony and 25 m seawater samples from 16S rRNA (A, B) and *nifH* (C,D) genes. Curves were produced using the vegan rarecurve function (http://CRAN.R-project.org/package=vegan).
