# Supplementary material for: Microbiome of Trichodesmium Colonies from the North Pacific Subtropical Gyre
Source: Front Microbiol. 2017 Jul 6;8:1122. doi: 10.3389/fmicb.2017.01122 (PMC5498550; doi:10.3389/fmicb.2017.01122)
Supplement: Supplementary file 8 [file Image2.pdf]

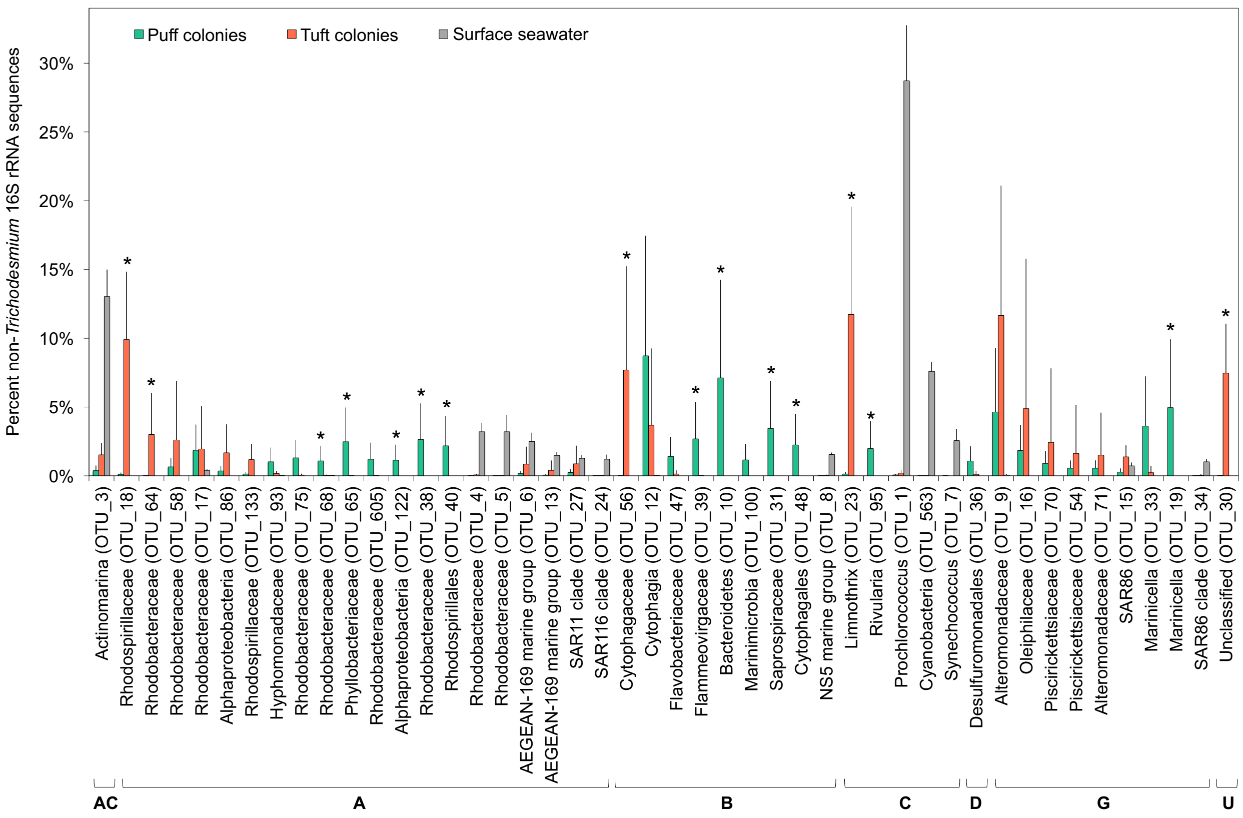


**Figure S2:** Dominant OTUs from the partial 16S rRNA dataset. The percent of non-*Trichodesmium* 16S rRNA gene sequences from puff colony, tuft colony, and surface seawater samples from OTUs containing an average of >1% of sequences for puff or tuft colony samples are displayed; this represents 64%, 77%, and 14% of non-*Trichodesmium* 16S rRNA sequences from puffs, tufts, and seawater samples, respectively. Brackets show OTUs classified as Actinobacteria (AC), Alphaproteobacteria (A), Betaproteobacteria (B), Cyanobacteria (C), Deltaproteobacteria (D), Gammaproteobacteria (G), and unclassified taxonomy (U). Error bars represent standard deviations of puff samples (n=7), tuft samples (n=5), and surface seawater samples (n=4). Asterisks denote a significant difference between puff and tuft samples ($p <$ 0.001, Welch Two Sample t-test with Bonnferroni correction for multiple comparisons).
