# Supplementary material for: Microbiome of Trichodesmium Colonies from the North Pacific Subtropical Gyre
Source: Front Microbiol. 2017 Jul 6;8:1122. doi: 10.3389/fmicb.2017.01122 (PMC5498550; doi:10.3389/fmicb.2017.01122)
Supplement: Supplementary file 9 [file Image3.PDF]

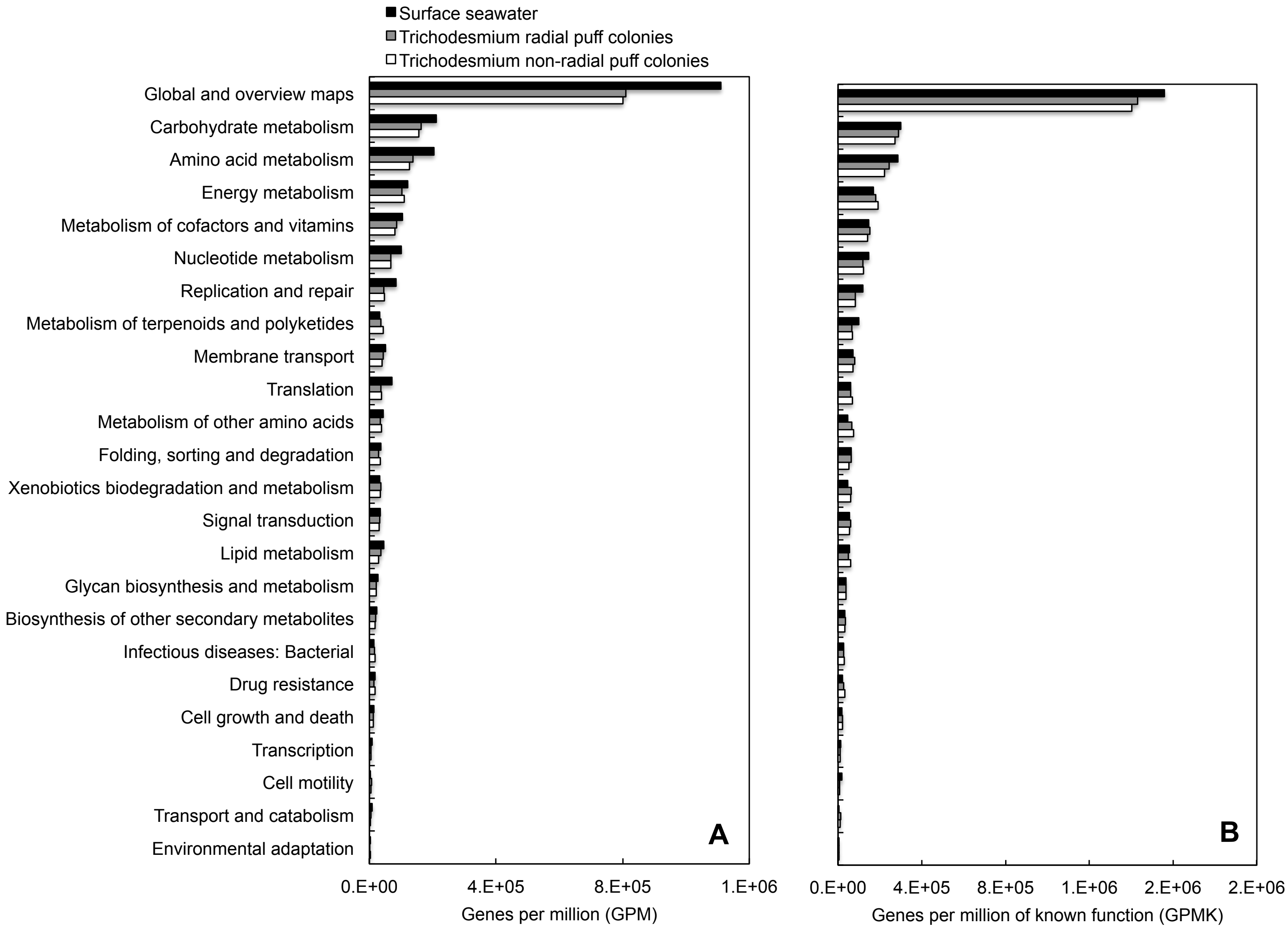

Figure S3: KEGG categories including summed KO from *Trichodesmium* colony and surface seawater samples using GPM (A) and GPMK (B) normalization methods.
